# Medical Versus Surgical Means of Diagnosis and Treatment of Gastrointestinal Diseases

**Published:** 1912-10

**Authors:** Anthony Bassler

**Affiliations:** New York City


### Medical Versus Surgical Means of Diagnosis and Treatment of Gastrointestinal Diseases.

### The Burning Question in Medicine.

By ANTHONY BASSLER, M.D.
New York City

MARCUS Aurelius, the wisest of Roman Emperors, died in 180. Before then he wrote: "Remember that all is but opinion, and all opinion depends on the mind. Take thine opinion away, and then as a ship that hath stricken in and within the arms and mouth of the harbor, a present calm; all things safe and steady; a bay not capable of any storms and tempests.'" XII, 16.

Accepting these words on opinion as true today, the situation in the subject considered tonight shows that we are still far at sea and to a great degree rolling tempestuously about. The internists and stomach specialists have had their opportunity for many years, and to great extent failed through omission. The surgeons have recently come forth, and also to a great extent failed but through commission. The general practitioners who man the deck and who have the deepest interest in the sick passengers, are all over it and nowhere at the same time according to the patient and as the consultents are inclined. At one time they are pro-surgical and rallying with the surgeon, and at another promedical and with the internist. Every general practitioner who has had enough experience with these difficult-to-fathom cases and been active in cooperative practice with them, will tell you of recent cases that he took to internists and stomach specialists and they failed him, and others which he delivered over to surgeons and they failed him too. In the same breath, he will relate apparently surgical cases in which he was glad that no operation had been done, and not a few in which he was thankful that it had been performed after having had long and bitter discouragement with the internist and stomach specialist. If my work was that of catering to a general family practice, on this subject, I would stand in the centre of the deck and huddle my patient up close beside me with both arms around him and keep perfectly still until my own reasoning directed me the way, or some diagnostician, whatever his classification, advancing reason and logic on the case would show me how to turn with him.

It is generally agreed that we are striving to bring practical medicine to a more definite basis, a mighty difficult, tedious and often discouraging thing to accomplish in only one of the myriad of details in this subject. In this, the surgeon who plays to the galleries in his rantings against stomach specialists accomplishes but little excepting selfish personal ends. To the few cases in a hundred of all chronic disorders manifesting symptoms in the abdomen he bases his wrath upon, the general practitioners, internists, stomach specialists, and even the surgeons themselves, see 95 that do not require operation. It is well enough for him to say to the stomach specialists "come into the operating room and learn of the criminal mistakes you have made," but the stomach specialists can truthfully say to him "come to my clinic and attend in my office and I will show you twenty cases with symptoms similar to the one you have operated upon that are getting well without operation."

The effect of these five per cent. of all cases, assisted as it is by the freedom of danger today in exploratory incisions, makes the surgeon radical in his views, and my observation has been that the less the surgeon knows and can accomplish, and for other personal reasons, the more radical he is. The time will never come when the majority of the people will submit to operation for diagnosis. Some of them will, but many times more feel that we know more about diagnosis than we really do, and will hesitate a long time, try different practitioners, and perhaps set their minds sternly against it. If you present them with a definite diagnosis, however, and this is an operative condition, the majority will submit early, but for exploratory incision, I suppose I voice the troubles of you all when I say that I have mine.

There are none, it is true, who deserve greater censure than that most numerous type of stomach specialist we have in the large cities today. As I view it, a stomach specialist must be a good general practitioner, an excellent internist, a consulting surgeon, a skilled laboratory man and radiographist, a neurologist, ophthalmologist and others, and be a dog for work on every case that comes to him, and this combination represents as rare a type of man as can be found in medicine anywhere. It is this ultraconservative, poor diagnostician type of stomach specialist who has temporized with the cases of chronic gastric and duodenal ulcer, gall stones, chornic appendicitis and others, and who does not make a diagnosis of gastric carcinoma until the growth is the size of a base-ball. I have often said, that the serious cases stand a better chance in the hands of good general practitioners of medicine than in his, although at times he may be brilliant with a few.

Good surgery of the abdomen is now only about seven years old. At first it was solely for therapeutic purposes, and of late it has become diagnostic also. For therapeutic purposes it was, is today, and always will be based right, for in proper cases it can do what medicine never will be able to. For diagnostic purposes as it is today, it is not based right and represents lack of medical knowledge, over ambition and deficiency of clinical work in fathoming cases. There are some cases which can only be diagnosed by that means, but these are decidedly fewer than are being operated upon today. Because of the bright results attained by surgery, the encouraging and sometimes questionable statistics, the therapeutic and I hope to a constantly growing less extent the diagnostic deficiencies of the internists and stomach specialists, the surgeons have been crowding the internists to the wall and offering operation as the alternative. In medicine, the tendency of its leaders is to cover up mortality rates and mistakes, and only present the bright results. This is more true today of surgery than of medicine, because we rarely learn of the surgeon's error, and the patients live on for some time beyond that of the internists so that they can come to us. Since all sides have lost sight of the necessity for the existence of special diagnosticians in this field who straddle both the medical and surgical aspects of cases, and since we are more familiar with the deficiencies of our internists and stomach specialists in the past, let us consider what any one can see on the surgical side.

In the last five years, my associates and myself have examined in the clinics over six thousand cases giving symptoms referable to the abdomen. Ninety-one per cent. of these had had symptoms for six months or longer. Forty-three per cent. had been operated upon at some time in adult life for something. In fourteen per cent. laporatomy scars were visible, and in eleven per cent. these represented operations for some condition in which there had been abdominal symptoms. This eleven per cent. was made up by 570 cases. In 460 digestive symptoms persisted after the operation as before and many had grown worse after it. Of the 110 cases that were benefitted, 94 were operated upon after more or less definite diagnosis had been made, and in 86 the patients told of what was found or done. Of the 460 cases that were benefitted but very little, none at all, or made worse, the patients went on the table with suggestive degrees of definite diagnosis in only 73 cases. Leaving these out of consideration, we may say that needless exploratory incisions and operations were performed in 387 out of 460 cases, and, most interesting, very few of these patients knew anything about what was or was not found at operation. Putting the rate of mortality from exploratory incision and general anesthesia down to one and a half per cent., conservatively, six individuals have ceased to exist in the meantime. In these 387 cases, the operations were performed in 17 of the New York, Bronx and Brooklyn hospitals, and the great majority by the best men we have here in surgery today. I feel that the time has come to call a halt on operations to see if something may be found, rather than operations on some reasonable indications for them.

Knowing that surgery is not a "cure all' for stomach diseases as some of us would want all to believe, what have the major of these mistakes been due to? During the last years gastroenterostomy has become a common operation, and still the only conditions in which it is helpful are stenoses to the onward flow of chyme in the stomach and chronic duodenal ulcer, and these comprise but few of all cases that are seen. Even though it is true that the duodenal ulcers are prone to chronicity from the start, why is it that at autopsies ten ulcers of the stomach are found to one of the duodenum, and that at operation more duodenal are met with than gastric? Is it only because they are not diagnosed, or do the majority of them heal at some time? I think that both are true, and wish to state that if more of them were diagnosed and treated by three weeks bed and diet and six months dieting afterward, that about one-fourth of all would heal which are today meeting with surgery therefor. Since four-fifths of all gastric ulcers are in the lesser curvature in the posterior wall of the stomach—a surgical inaccessible place—and since gastroenterstomy in a curative way is helpful in only a small proportion of them and partial gastrectomy is such a serious matter, should every chronic gastric ulcer be operated upon? This is a moot question, for most should be and others should not.

The strongest argument in favor of generous surgery is in the prevention and saving of life from malignant disease. According to some surgeons, old ulcers are the basis of this in the majority of instances. Careful autopsies on those who have died of malignant disease of the stomach do not prove this to be true. If carcinoma can develop in a gall-bladder which has no stones, the head of the pancreas which is well protected from trauma, in a uterus which is freely suspended and only the erosion of the cervix of which can be taken as a probable predisposing factor, in the distensile and simple mucous membrane of the rectum, and in the breast of virgins who have not even had a definite trauma, then we have a right to believe that it may develop in the stomach too without an ulcer having first been there. The usual step in the process of degeneration of epithelial tissue is hyperplastic before it becomes atrophic, and degeneration may take place from general as well as simple local causes. Such hyperplasia means small round cell infiltration, and if it were not for collection into carcinoma nests you could not tell the difference between the tissue of simple inflammation and malignant disease, for the cells of both look just alike. May not then such local inflammation in susceptible individuals, which can come from causes other than ulcer, continue in the cell proliferation with the development of malignant disease? I think so, for cancer does not kill by the disease alone, it kills because of pressures, hemorrhages, perforations, and deadening interference with the functions of important organs.

In Mayo's review of 266 partial gastrectomies for cancer of the stomach, the condition of 191 of which was known three years and over after operation, 38 were alive and well. Averaging all of his statistics of over three years after operation, one-fourth of the cases whose condition was known are reported alive and well. This is a strong argument in favor of generous surgery, because the majority of these patients have no symptoms of disorder until late and thus do not come under observation in time, and, with many of those that do, the medical men do not diagnose them—although at least a diagnosis of 'surgery indicated' can be made in most of these if careful detail work is done. Now, if the case does not come under the internists or stomach specialists' attention, either because they have had no symptoms or been temporized with too long by general practitioners, the blame cannot be put on the internists or stomach specialists, but in those that do, they are then guilty of criminal neglect if immediate surgery is not encouraged. Mayo himself said: "It is my intention to call attention to two important indications for operation, which, if observed early, will be the means of securing a number of patients in time to benefit them. 1. Food remnants found repeatedly in the stomach after twelve hours, when taken in connection with the clinical history, should indicate a surgical consultation and, in the large majority of cases, this special investigation will lead to an exploratory operation. 2. The finding of a movable tumor in the pyloric end of the stomach has a surgical significance which cannot be over estimated. It is a great mistake to suppose that the presence of a movable tumor is indicative of a hopeless condition." To these I would add, the peristaltic wave jumps and localized thickenings in the pyloric end of the lesser curvature as seen by X-ray, and the special chemical tests of the stomach contents and feces. Along these four lines of investigation and careful study of the histories, most all of the cases can be picked out, and then the incisions made would be less often an exploratory incision than the first step in the performing of a partial gastrectomy.

That gall-stones are found present in ten per cent. of all autopsies and indigestion is so common, are not arguments enough in favor of general exploratory incision to make diagnoses of cholelithiasis. In the cases of recurring attacks of biliary colic with or without subsequent jaundice and other suggestions, the diagnosis is usually easy, but it is regarding the cases in which diagnosis is more difficult that I would speak. A decided constant tenderness in the gall-bladder with a chronicity of symptoms must be present before I would consider operation in these cases; and the same is true with all of the non-empyemic cholecystitic conditions. We may all agree that gall bladder cases which give local, gastric or general symptoms are always operative cases, but I feel that almost everyone of those operated upon and in which gall-bladder pathology or stones are not found is wrong, and many of these are occurring every day which would not be so if more care were taken in the examinations.

Agreeing that acute hemorrhagic and suppurative pancreatitis are operative conditions, the diagnosis of either one is not so difficult. The symptoms of these pancreatic conditions, and perforations of the stomach, duodenum and gall bladder, and acute intestinal obstructions are always acute and severe enough to warrant immediate operation even when a diagnosis of one from the other cannot be made in advance. But why are so many exploratory operations being performed in which only chronic pancreatic disease is disclosed when careful work in feces and other tests will make the diagnoses just as well and when surgically nothing can be done for them?

A word now about peritoneal adhesions (not, for instance, the thick organized bands which run from the pelvis to the upper abdomen and those binding the hollow viscera into stenoses, which are always operable conditions), but just the short and easily broken down adhesions. Does an operation in which only adhesions are seen and separated and nothing more found to be done justify the performing of it. I do not think so, for my experience has been that no benefit comes from these operations and often decided harm is done.

Now that always interesting subject of appendicitis, about which in the years gone by so much has been said and written and for which so many operations have been done. There are some of us who were active in practice at the time the gridiron and short incision operation became popular and who are still active in medicine. Not one of these men would do other than worship at the shrine o fthis operative therapy on all true appendicitic conditions, and who would not agree to have his own and his patient's appendix removed at once when bona fide disease of it was present. But as the frequency of appendectomy has developed today, may we not look with a certain degree of question on the many appendices that are being removed, and ask was the operation really necessary? A week's attendance in the operating room of any of our great hospitals which have a large and active service side will display an accumulation of removed appendices which are a study in themselves. In former days much more so than now, we saw adhesion, strictured, bulbous, concretion, septic and gangrenous cases operated upon as the routine. Now these distinctly pathological appendices are by no means so common in the collection as formerly, as one can easily see from the clinical study of the cases operated upon, the operative findings, and the gross and microscopical studies of the specimens. Today, it seems sufficient for the surgeon's conscience to have removed one only short and stumpy, one long angle-worm like, one curled like a pug dog's tail or laying retrocecal, one having a firm feel or quite relaxed, one with slight degrees of irregularity in the lumen or slight thickenings of the mucous membrane, and so forth. Mind you, I believe that every case of true appendicitis should be operated upon and at once, but the chronic gastric cases not due to appendicular disease wherein the appendix is removed because it can in some cases be a cause of gastric symptoms is going too far into needless surgery. A careful gross and minute pathological study of the appendices of a large number of individuals who have died from disease other than those of the abdominal structures, has proven to me that but few living and well individuals have perfectly normal appendices, and yet would it be warranted to remove every one of these, and force upon the public the dangers of post-anesthetic and post-operative neurasthenia? I doubt that there is reason enough of a prophylactic nature in the prevention of serious appendix disease to operate primarily upon all such appendices as these, because but few of them all ever would develop a serious trouble. We must have direct or at least good reason to suppose that definite appendicular disease exists before an appendectomy is justiifiable, and cases should not be operated upon only on the chance that this may be so. Of course, when operation has been performed for some condition other than appendicitis it is well to remove the appendix as a prophylactic measure, but here the incision has been made and that justifies the appendectomy.

In this series of 387 individuals, appendectomy had apparently been performed 197 times, and yet they were no better, and many times worse because of it. If there is any one thing that shows the diagnostic shortcomings and liberal hands of the surgeon it surely was this group. Many times in other cases, the appendix was removed only a short time before and a chronic gastric or duodenal ulcer, gall-stones, etc., present at the time of its removel, left untouched. There were 9 cases in the group in which an appendectomy had been performed inside of a few months before we saw the patients with large sized hard nodular masses in the upper abdomen and all the symptoms of undoubted instances of advanced cancer of the stomach, and these were exactly the cases in the most numerous instance in which the surgeons rail hardest against the internists and stomach specialists. ' Plainly they are falling short too, or are developing a mortality in the search for a few of these conditions.

The statistics of surgery of the abdominal cavity will never be complete until they include the post-anesthetic and post-operative neurasthenias and psychoses. Every stomach clinic in the world is overrun with a type of individual who is more neurasthenic than otherwise, and these with the primary atonies and prolapse cases are the ones who made up most of the 387 cases that were needlessly operated upon. To operate on a primary neurasthenia is positively unpardonable unless there is an intercurrent condition that warrants it to save his life or keep him from added suffering. In 69 of these 387 cases it was a fair clinical conclusion to arrive at that neurasthenic symptoms which were not present before had developed as a direct consequence of the operation. Of the 318 remaining, the individuals had become more distinctly neurasthenic in apparently 205 instances, and the 113 remaining it either was not so or there was question enough about it not to consider this in a definite way. Some of these had been operated upon as many as six times, and more pitiful was the fact that with many of them they were ready for as many more laporotomies as they would or could fall into the hands to do for them.

If you take a thousand cases that come with stomach trouble, you can learn that from diagnostic and therapeutic standpoints they are wholly medical in 95 per cent., and medical and surgical in 5 per cent. Thus, on this subject the internists and stomach specialists cannot be ultra-medical or the surgeons ultra-surgical. Every case should be medical before it is surgical, and the gap between the general practitioner and the surgeon should be filled by a new type of stomach man who is strong in diagnosis and inclined medically or surgically as the case may prove itself to be. The mistakes and shortages of the internists and stomach specialists mostly have been due to lack of work, and those of the surgeons to overwork. We cannot advance this part of medicine excepting on the basis of pathological diagnosis, which is neither medical nor surgical alone. Both sides must work in conjunction more than they do today, and the internists and stomach specialists must give better and more diagnoses to the surgeons than they now do, or at least say that exploratory incision is indicated, and surgeons should not operate unless there is some strong suggestion or logical reason for doing so.

I have tried in this paper to express some of my opinions based upon clinical observations. In the writing of it only the kindliest of motives have moved me although it may appear that some hidden attack is contained in my words. I cannot feel that any of us are so narrow and self satisfied that we would not place the patient's interests above our own. Criticisms and suggestions in medicine should not be born of enmity and antagonism. With a more general sincerity, the medical men will make a better progress through surgery and the surgeons through medicine, for on this subject we cannot fail to know the right when so much is at stake.

